# Telemedicine and Home-Based Treatment of COVID-19 in Resource-Limited Countries. Report of 3 Cases

**DOI:** 10.5152/eurasianjmed.2021.20227

**Published:** 2021-06

**Authors:** Killen H. Briones-Claudett, H. Briones-Claudett Mónica, Killen H. Briones-Zamora, Diana C. Briones-Márquez, Andrea Icaza-Freire, Michelle Grunauer

**Affiliations:** 1 University of Guayaquil, Faculty of Medical Sciences, Guayaquil, Ecuador; 2 Physiology and Respiratory Center Briones-Claudett, Guayaquil, Ecuador; 3 Intensive Care Unit, Ecuadorian Institute of Social Security (IESS), Babahoyo, Ecuador; 4 Universidad Espíritu Santo, Samborondón, Ecuador; 5 Universidad San Francisco de Quito School of Medicine, Quito, Ecuador; 6 Department of Critical Care, Hospital de los Valles, Quito, Ecuador

**Keywords:** COVID-19, Telemedicine, Health Services

## Abstract

Ecuador, despite having taken aggressive and early measures to stop the progression of the pandemic (COVID-19), ended up becoming an epicenter of the pandemic in Latin America, and with the collapse of its health care system. The authors describe three patients who had confirmed COVID-19 and met the criteria for hospital admission but could not be assigned a hospital bed in a resource-limited country. The patients included a 72-year-old male, an 82-year-old female, and a 56-year-old male. They typically presented with fever, dyspnea, loss of taste and smell, diarrhea, and abdominal pain. Oxygen saturation during the initial evaluation ranged from 80–89%. Laboratory results reported lymphopenia and neutrophilia, with leukocytosis in two patients. Inflammatory markers were also elevated for all three patients. CT scan findings showed bilateral ground-glass pulmonary opacities. SARS-CoV-2 was confirmed in all three patients by real time polymerase chain reaction (RT-PCR) testing. Home-based treatment was established. At the time of writing this report, all patients remain asymptomatic and with negative COVID-19 testing. Telemedicine and home-based treatment were essential assets in the care of these severely ill patients living in a low-resource setting where not all patients who have criteria to be admitted into the hospital are able to find a place in a collapsed health care system.

## Introduction

The first confirmed case of acute respiratory syndrome coronavirus 2 (SARS-CoV-2) in Ecuador was diagnosed on February 29, and, as of April 19, there have been 9,468 confirmed cases with 474 confirmed deaths and 817 probable coronavirus deaths.^1^ With 17 million citizens, Ecuador is the country with the highest death toll in the region. By far, the most affected province in the country is Guayas, where more than 65% of the reported cases are clustered. Furthermore, in the first 15 days of April 2020, the province of Guayas reached a count of 6,703 deaths from various cases. When compared with the same period in 2019, an increase of 5,700 deaths was evidenced.^2^

Here, we describe our experience with three cases of COVID-19 positive patients, who were unable to be admitted into the hospital setting because the capacity of the health care system has been surpassed at this point due to the inability to hospitalize critical patients and required out-patient management.

## Clinical and Research Consequences

These patients were unable to receive prompt and timely care by local hospitals owing to exceeded capacity. Endurance of long waiting hours before medical assistance, especially in older adults with preexisting comorbidities, is believed to worsen outcomes. We therefore believe that telemedicine can provide convenient access to medical care in this scenario.

Home-based treatment was established according to guidelines provided by the Ecuadorian health system and current clinical trials. 3

We report the original results of the first three patients who were treated by our team using a telemedicine home-based care approach during the COVID-19 pandemic in Guayas. The summary of the characteristics and laboratory tests of the patients (see table 1)

This approach was very challenging and emotionally exhausting for everyone involved. Our patients met the criteria for hospital admission, however, not a single bed was spared during the collapse of the health system in the city of Guayaquil from 03/27/2020 to 04/15/2020. Many ethical concerns were raised by us, as evidence-based treatments are still lacking. Furthermore, we were aware that our patients needed hospitalization. At the same time, we had to consider benefit versus harm, autonomy, and our context, as well as try to attenuate the impact of distributive justice in our country. Looking back, we think that we made the best possible decisions we could in extremely difficult circumstances. Our patients are alive and well, even though we can’t state if our treatment, a component of our treatment, if any, had effect in the outcome.

Based on the outcomes described in these three patients, it is not possible to determine whether this treatment is optimal for patients with coronavirus disease (COVID-19). However, it remains an important consideration in low-resource settings where not all patients who meet the criteria for hospital admission are able to find a place in a collapsed health care system. Telemedicine and home-based treatment were essential assets in the care of these severely ill patients.^4^

Telemedicine and home-based treatment were pivotal during the pandemic in the province of Guayas and offered the possibility of continuity of care for severely ill patients who could not be admitted to hospitals owing to the lack of infrastructure. In response to the pandemic, the provision of telemedicine in a home-based care setting seemed useful in preventing and identifying progressive deterioration in a timely manner in deprived settings such as ours .^5^

## Conclusion sections

Telemedicine could play a key role in responding to the attention of those severely ill patients who are living in a low-resource setting where not all patients who meet the criteria to be admitted into the hospital are able to find a place in a collapsed health care system. A home-based treatment can be essential and timely in the management of these patients.

## Figures and Tables

**Table 1:** Characteristics of Patients

Characteristic	Patient 1	Patient 2	Patient 3
Age (years)	72	82	58
Gender	Male	Female	Male
Past medical history	Allergic rhinitis Former smoker for 15 years	Former smoker of 40 pack years COPD with multiple hospitalizations for pneumonia and exacerbations Diabetes mellitus for 12 years Coronary artery disease with angioplasty and stent placing Hypertension	None
Presenting symptoms	Fever, pharyngitis, productive cough with white sputum, thoracic pain, dyspnea, arthralgias, headache, loss of smell and taste sensations, diarrhea	Fever, dyspnea, loss of smell and taste sensations, abdominal pain, diarrhea	Fever, dyspnea, loss of taste and smell sensations, headache, diarrhea, arthralgias, myalgias
RT-PCR testing for SARS-CoV-2	+	+	+
Ambient air oxygen saturation (%)	88	80	89
Laboratory findings			
White cell count (per mm^3^) (normal range 4400 to 10300)	10,540	8,190	7,920
Differential count (per mm3)			
Total neutrophils (normal range 1,780 to 5,380)	9,430	7,050	6,771
Total lymphocytes (normal range 1,180 to 3,740)	630	730	498
Total monocytes (normal range 250 to 710)	240	330	609
*PCR (mg/dl) (normal range 0 to 5)	37.69	12.52	113.41
Ferritin (ng/ml) (normal range 30 to 400)	831.5	264.8	526.1
**IL-6 (pg/ml) (normal range 0 to 6.5)	59.9	36.1	32.7
D-dimer (mg/l) (normal range 0 to 1.9)	2.5	7.8	0.25
Procalcitonin (ng/ml) (normal range < 0.046)	1.39	0.13	0.22
Myoglobin (ng/ml) (normal range 28 to 72)	88.40	92	Not performed
Findings of Tomography			
Ground-glass pulmonary opacities	+++	+++	+++
Condensation areas	++	++	+
Crazy paving	+	−	−
Solitary nodule	++	++	++
Prescriptions	Supplemental oxygen, azithromycin, cefadroxil monohydrate, nitazoxanide, chloroquine, lopinavir/ritonavir, acetaminophen, fondaparinux, and tocilizumab	Supplemental oxygen, azithromycin, chloroquine, nitazoxanide, fondaparinux, simvastatin	Supplemental oxygen, azithromycin, cefadroxil monohydrate, chloroquine, lopinavir/ritonavir, fondaparinux, simvastatin
Survival	Yes	Yes	Yes

* PCR = C-reactive protein

** Interleukin 6 = IL-6
